# Assessment of the Feasibility of Objective Parameters as Primary End Points for Patients Affected by Knee Osteoarthritis: Protocol for a Pilot, Open Noncontrolled Trial (:SMILE:)

**DOI:** 10.2196/13642

**Published:** 2024-06-28

**Authors:** Dumitru Emanuel Dogaru, Serban Rosu, Dionisio Franco Barattini, Simone Guadagna, Luca Barattini, Bogdan Andor

**Affiliations:** 1 Department of Orthopaedics - Traumatology Victor Babeş University of Medicine and Pharmacy Timisoara Romania; 2 Clinical Research, Oral and Maxillofacial Surgery Victor Babeş University of Medicine and Pharmacy Timisoara Romania; 3 Opera CRO, a TIGERMED company Timisoara Romania; 4 TIGERMED Italy Genova Italy; 5 Profesor Universitar Doctor Teodor Șora Research Centre Victor Babeş University of Medicine and Pharmacy Timisoara Romania

**Keywords:** pilot trial, feasibility study, knee osteoarthritis, hyaluronic acid, outcome assessment, osteoarthritis, ultrasonography, knee, pain

## Abstract

**Background:**

Osteoarthritis (OA) is a disabling condition that affects more than one-third of people older than 65 years. Currently, 80% of these patients report movement limitations, 20% are unable to perform major activities of daily living, and approximately 11% require personal care. In 2014, the European Society for Clinical and Economic Aspects of Osteoporosis and Osteoarthritis (ESCEO) recommended, as the first step in the pharmacological treatment of knee osteoarthritis, a background therapy with chronic symptomatic slow-acting osteoarthritic drugs such as glucosamine sulfate, chondroitin sulfate, and hyaluronic acid. The latter has been extensively evaluated in clinical trials as intra-articular and oral administration. Recent reviews have shown that studies on oral hyaluronic acid generally measure symptoms using only subjective parameters, such as visual analog scales or quality of life questionnaires. As a result, objective measures are lacking, and data validity is generally impaired.

**Objective:**

The main goal of this pilot study with oral hyaluronic acid is to evaluate the feasibility of using objective tools as outcomes to evaluate improvements in knee mobility. We propose ultrasound and range of motion measurements with a goniometer that could objectively correlate changes in joint mobility with pain reduction, as assessed by the visual analog scale. The secondary objective is to collect data to estimate the time and budget for the main double-blind study randomized trial. These data may be quantitative (such as enrollment rate per month, number of screening failures, and new potential outcomes) and qualitative (such as site logistical issues, patient reluctance to enroll, and interpersonal difficulties for investigators).

**Methods:**

This open-label pilot and feasibility study is conducted in an orthopedic clinic (Timisoara, Romania). The study includes male and female participants, aged 50-70 years, who have been diagnosed with symptomatic knee OA and have experienced mild joint discomfort for at least 6 months. Eight patients must be enrolled and treated with Syalox 300 Plus (River Pharma) for 8 weeks. It is a dietary supplement containing high-molecular-weight hyaluronic acid, which has already been marketed in several European countries. Assessments are made at the baseline and final visits.

**Results:**

Recruitment and treatment of the 8 patients began on February 15, 2018, and was completed on May 25, 2018. Data analysis was planned to be completed by the end of 2018. The study was funded in February 2019. We expect the results to be published in a peer-reviewed clinical journal in the last quarter of 2024.

**Conclusions:**

The data from this pilot study will be used to assess the feasibility of a future randomized clinical trial in OA. In particular, the planned outcomes (eg, ultrasound and range of motion), safety, and quantitative and qualitative data must be evaluated to estimate in advance the time and budget required for the future main study. Finally, the pilot study should provide preliminary information on the efficacy of the investigational product.

**Trial Registration:**

ClinicalTrials.gov NCT03421054; https://clinicaltrials.gov/study/NCT03421054

**International Registered Report Identifier (IRRID):**

RR1-10.2196/13642

## Introduction

### Background

Osteoarthritis (OA) is a joint disease that causes inflammation and reparative bone response. It is a highly prevalent condition, affecting over 100 million people worldwide, and ranking among the top 5 most disabling conditions. More than 50% of the global population older than 65 years exhibit radiographic evidence of OA in at least 1 joint, indicating a high incidence of this disease. OA affects both sexes equally, although it seems to be more prevalent in men younger than 45 years and in women older than 45 years. Currently, 80% of individuals affected by OA experience movement limitations, and 20% are unable to perform important daily activities. Furthermore, 11% of the affected population requires personal care.

In OA, the articular cartilage undergoes repetitive inflammation due to focal loss or erosion, leading to joint space narrowing or subchondral sclerosis. OA can cause hypertrophy of osteoblastic activity or reparative bone response, which is known as osteophytosis. This can result in pain, immobility, and often disability. OA symptoms, such as joint pain, stiffness, and muscle weakness, are significant risk factors for mobility limitation and can negatively impact the quality of life (QoL) of affected individuals [1]. According to the literature, individuals with genetic predisposition, obesity, aging, or previous joint injury are more likely to develop OA at an earlier age. This disease is primarily caused by repetitive mechanical loads, age, and high levels of inactivity [2,3]. Physiologically, it is characterized by the loss of cartilage covering the joints, resulting in direct bone-to-bone contact [4]. The symptoms may vary greatly among individuals. While some patients may experience incapacitation due to the disease, others may be able to perform normal activities with little or no pain, despite the severity of the condition, as indicated by x-rays. Knee OA is commonly observed in individuals who engage in intense physical sports that require significant strain and loading on their joints, such as American football, soccer, rugby, and gymnastics. A prior injury is a major indication for future development of the disease. Obesity in the upper extremity is often associated with a major cause of knee OA, which results in heavy weight-bearing on the knee [5].

### Rationale

In 2014, the European Society for Clinical and Economic Aspects of Osteoporosis, Osteoarthritis and Musculoskeletal Diseases (ESCEO) recommended using symptomatic slow-acting drugs for OA (SYSADOAs)—with the addition of paracetamol for short-term rescue analgesia when needed—as the initial pharmacological treatment for knee OA [6]. SYSADOAs exhibit excellent safety and tolerability. They consist of natural compounds that include repeating disaccharides, such as glucosamine sulfate, chondroitin sulfate, hyaluronic acid (HA), diacerein, and avocado-soybean unsaponifiable [7,8]. In recent years, extensive research has been conducted on HA, particularly its efficacy in treating mild to moderate knee OA through intra-articular (IA) administration. A meta-analysis of over 137 studies, including randomized clinical trials (RCTs), provided evidence of its effectiveness [9]. Following our experience in daily orthopedic clinical practice with the administration of both IA and oral HA, we have recently focused our research activities in OA on the use of oral high-molecular-weight HA (HMWHA). Although there is direct evidence that ingested HA reaches the knee joint in human-relevant preclinical mammalian models [10,11], it has not yet been determined whether HA is bioavailable in humans after oral administration. RCTs on oral HA administration are limited and inconsistent. Recent reviews have shown that while oral HA has been effective in relieving knee pain in various clinical trials without side effects, the heterogeneity of the efficacy end points strongly limits the generalization of these results [12]. Additionally, it has been observed that many of these trials used subjective parameters to measure pain, such as the visual analog scale (VAS), or specific scores for OA outcomes, such as the Western Ontario and McMaster Universities Osteoarthritis Index (WOMAC) or the Knee Injury and Osteoarthritis Outcome Score (KOOS). In contrast, objective measurements, such as isokinetic dynamometry and ultrasonography, have been infrequently used and have shown several inconsistencies. With these considerations in mind, we conducted a thorough analysis of the existing medical literature [13] and decided to plan a double-blind RCT to test an oral formulation of HA in a calculated sample size of 80 patients with OA. The objective of the proposed study is to test the hypothesis that oral administration of HMWHA for at least 4 months would (1) reduce knee pain at the end of the study period, (2) demonstrate a statistically significant difference compared with a placebo-treated control group, and (3) demonstrate the aforementioned improvement by both subjective (eg, VAS and QoL) and objective measures (eg, ultrasonography). Unfortunately, conducting an RCT in a large patient population may pose a significant risk to our investigative team given our limited resources. Performing a pilot feasibility study before conducting the main RCT may be a reasonable solution. This 2-step procedure can help to prevent dangerous consequences when initiating the main trial. In fact, it can evaluate the feasibility of the monthly recruitment rate, estimate the time and budget required for the RCT, and avoid potential bias in the selection of end points and their measurements [14].

**Figure 1:**
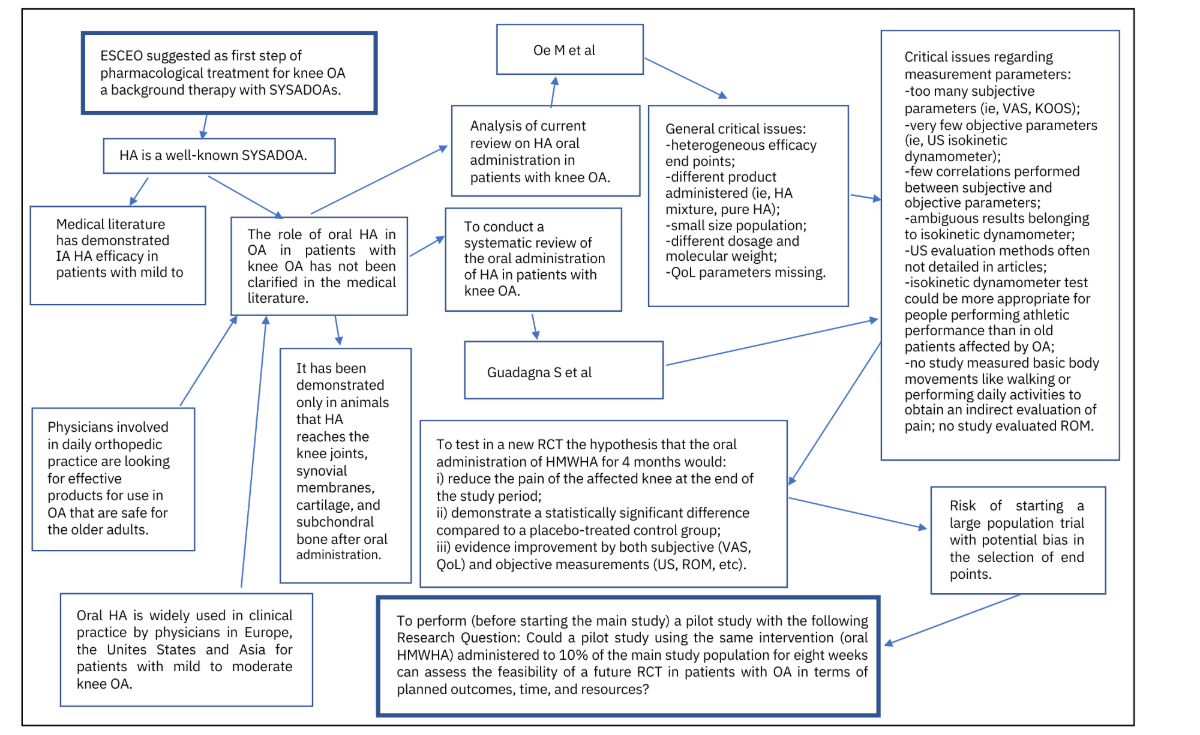
Mind map showing the rationale of the pilot study. ESCEO: European Society for Clinical and Economic Aspects of Osteoporosis, Osteoarthritis and Musculoskeletal Diseases; HA: hyaluronic acid; HMWHA: high-molecular-weight hyaluronic acid; IA: intra-articular; KOOS: Knee Injury and Osteoarthritis Outcome Score; OA: osteoarthritis; QoL: quality of life; RCT: randomized controlled trial; ROM: range of motion; SYSADOA: symptomatic slow-acting drug for OA; US: ultrasonography; VAS: visual analog scale.

The research question, as shown in Figure 1 [12,13], is whether a pilot study using the same intervention (oral HMWHA) administered to 10% of the main study population for 8 weeks can assess the feasibility of a future RCT in patients with OA in terms of planned outcomes, time, and resources.

## Methods

### Overview

The study team is composed of 2 investigators from the University of Medicine and Pharmacy “Victor Babes” in Timisoara, Romania, in addition to the nurses and study coordinators from the participating site (Centrul Medical Medicali’s, a private clinic specializing in orthopedics located in Timisoara, Romania). The Contract Research Organization (Opera CRO, a Tigermed company based in Romania) is selected directly by the team to handle logistics and study management, including document submission to the ethics committee (EC) and competent authority, project management, site monitoring, data management, and statistical analysis. The team choose the logo (Multimedia Appendix 1) and the study name :SMILE: (Syalox, Multicenter, Trial to Evaluate Knee OA) and prepare the protocol and all related documents, including the case report form (CRF), patient information leaflet, and informed consent form required for the feasibility pilot study. Before the project began, the study protocol was presented to River Pharma, a company based in Orio Litta, Italy, that manufactures a food supplement containing HA. The company provides the study team with the necessary products for both the pilot and main OA studies and partially funds the trials. The agreement between the parties stipulates that the sponsor will not be involved in the design, planning, collection, analysis, or interpretation of data in any future manuscript reporting the study results.

### Study Design

This is an open-label, noncontrolled, single-center pilot feasibility study.

### Ethical Considerations

The study protocol with the code OPRPH/0117/FS is approved by the local independent EC of Timisoara, Romania, the Comisiei Locale de Etica pentru cercetare stiintifica a Centrului Medicali, on December 12, 2017 (request # 012; Multimedia Appendix 2). Informed consent is obtained from each potentially eligible patient in accordance with the International Council for Harmonisation Good Clinical Practice (GCP) guidelines and the current version of the Declaration of Helsinki. Additionally, each included participant receives a patient information sheet that explains the trial’s goal and procedure. This document, like the informed consent form, is written in plain Romanian language and is preapproved by the local EC. The information sheet describes the nature of the administered food supplement, its common use in orthopedic clinical practice, and its safety profile. The information sheet outlines the number of visits, types of procedures, assessments, and measurements to be performed during the trial. This provides potential participants with a clear understanding of the risks, inconveniences, and benefits that may arise from their involvement. The sheet should be updated as new important information becomes available that may affect a participant’s willingness to participate or continue in the study. The investigators aim to inform potential participants that they are being invited to participate in a pilot feasibility study rather than a main study. Therefore, only future patients may benefit from the results of this study in terms of the efficacy of the product administered. In addition, the investigator should communicate the specific feasibility objectives of the study to the participants as well as the criteria for the pilot study to successfully lead to the main study. Participants are informed that their involvement in the trial is voluntary and that they may withdraw at any time while still receiving medical care. They are also informed that there is no compensation for their participation in the study and that the sponsor provides insurance coverage in the event of a trial-related injury. Potential participants are informed that their medical records would be reviewed by the sponsor, CRO personnel, and regulatory authorities in accordance with applicable laws and regulations, and that personal information would be collected and maintained in a confidential database. Written consent is obtained after the participants have been given sufficient time to review the information and ask questions. The procedure for obtaining informed consent requires that both the participant and investigator manually write and sign their names on 2 copies of the consent form after discussion. The investigator provides the participants with 1 copy of the signed informed consent form and 1 copy of the signed participant information sheet. The second copy of the original signed forms is kept in the on-site study file. The study adheres to the current General Data Protection Regulations (GDPR). Procedures for data collection and anonymization, as well as the explanation that data may be made available to other researchers in aggregate and anonymous forms, are detailed in a specific document (GDPR form) written in the local language and provided to the patient for approval and signature before the trial begins. The medical doctor in charge of the center is responsible for the treatment of patient data at the clinical site in accordance with the GDPR regulations.

This study protocol complies with the CONSORT (Consolidated Standards of Reporting Trials) statement for pilot and feasibility studies [15] and is registered at ClinicalTrials.gov under the identifier NCT03421054.

### Inclusion Criteria

Patients who meet the following criteria are eligible for inclusion: (1) any sex and age between 50 and 70 years and (2) symptomatic OA of the knee with mild joint discomfort for at least 6 months before enrollment, following American College of Rheumatology (ACR) criteria with history and physical examination [16] (participants diagnosed with bilateral knee OA are asked to identify the most affected knee at baseline [BL], and this knee is evaluated throughout the study period); (3) available confirmatory radiographic diagnosis (performed within the previous 6 months) with Kellgren-Lawrence score 2 at the knee joint evaluated [17]; (4) participants experienced pain for at least 15 of the 30 days before study entry; (5) signed informed consent; and (6) demonstrated compliance with all study requirements.

### Exclusion Criteria

Patients meeting any of the following criteria are excluded from the study: (1) any inflammatory arthritic condition (other than knee OA), fibromyalgia, multiple sclerosis, or autoimmune disorder; (2) oral corticosteroid treatment within 4 weeks before screening; (3) IA injections of HA or corticosteroids in the target joint within 3 months before screening; (4) participants who have taken anti-inflammatory or chondroprotective drugs (such as chondroitin sulfate, glucosamine, methylsulfonylmethane, HA, and diacerein) within 2 weeks before selection; (5) use of HA-containing nutritional supplements or cosmetics within the month before the study; (6) previous surgical treatment of the knee joint or require OA-related surgery (such as high tibial osteotomy or arthroplasty) or have complications that require hospitalization and surgical treatment; (7) significant injury to the target joint within the 6 months before screening; (8) participants who are following an energy-restricted diet for weight loss; (9) pregnant women, nursing mothers, or women of childbearing potential who are not using adequate methods of contraception; (10) participants with cardiovascular, hepatic, renal, respiratory, or hematologic disease, or any other medical or psychiatric condition that, in the opinion of the investigator, would impair participation or is likely to result in hospitalization during the course of the study; (11) participation in an interventional clinical trial within the past 30 days; and (12) presence of any clinically significant medical condition that, in the opinion of the investigator, precludes patient enrollment in the study.

### Discontinuation Criteria

Patients may withdraw from the study at any time on their request. The investigator may withdraw a patient from the study at any time for health reasons. In both cases, the investigator must explain the reasons for withdrawal on the CRF and the participant is considered to have withdrawn. Additionally, the study may be terminated by the investigator or sponsor for any of the following reasons: (1) insufficient participant enrollment at the center, (2) persistent or serious noncompliance with the protocol, and (3) noncompliance with GCP or regulatory requirements. If the trial is prematurely terminated or suspended for any reason, patients will be promptly informed and the EC and regulatory authorities must be notified.

### Recruitment

**Figure 2:**
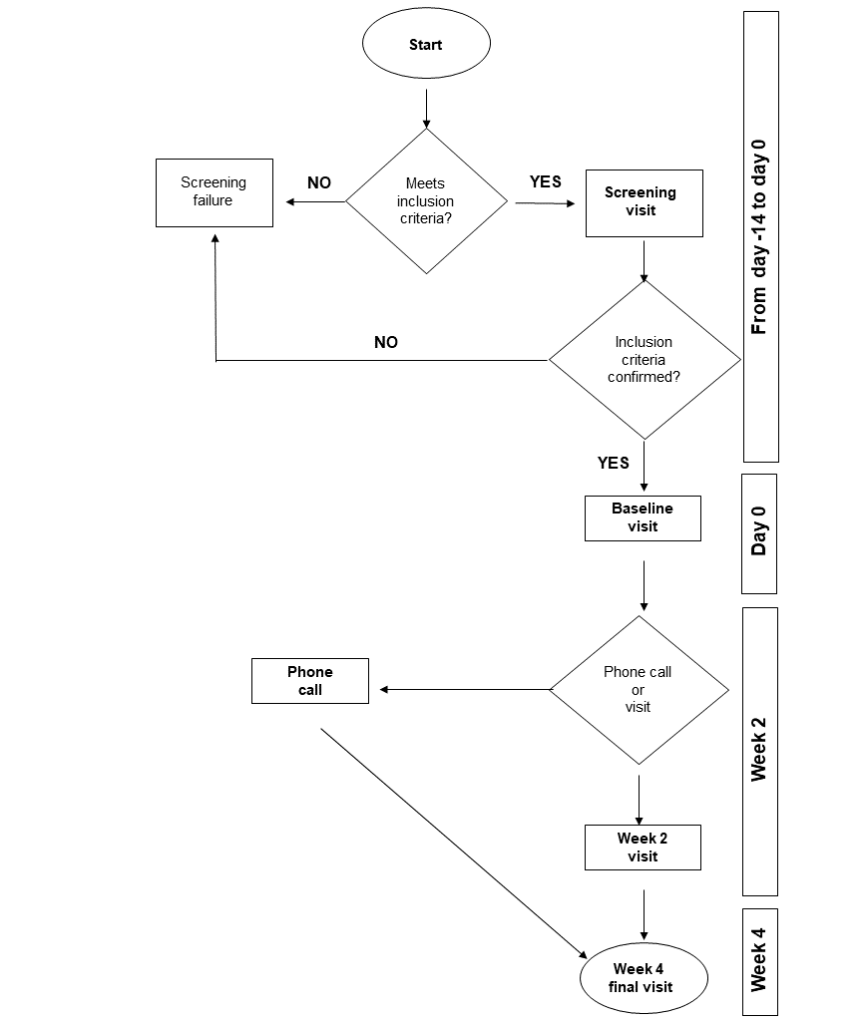
Flowchart of the pilot study.

The study team at the involved site, including the principal investigator, are doctors trained in GCP with over 10 years of experience in orthopedics or rheumatology. To prevent selection bias, patients should be consecutively enrolled. Therefore, all patients with knee OA and visiting the center from February 2018 (start of enrollment) to April 2018 (planned end of enrollment) should be screened, asked for informed consent, and checked for inclusion and exclusion criteria. The data for each patient must be recorded in the CRF. In cases where patients are screened but not enrolled owing to exclusion criteria or participation in another interventional study, the decision to exclude the patient must be clearly reported on the CRF. Our goal is to recruit at least 8 patients with knee OA according to the ACR criteria (Figure 2) from a single site over a 2-month period. These participants must meet the inclusion criteria outlined in the pilot study protocol, which will also be used in the future main study.

### Intervention

All patients will receive Syalox 300 Plus (River Pharma), a food supplement containing HMWHA. Although this product has been available in European countries for several years and has been used in the clinical practice by hundreds of orthopedists, only a few clinical trials [18] have been conducted to test whether this type of product with oral HA could complement the treatment of patients affected by OA. In this study, the investigational product (IP) is administered as 1 tablet daily for 8 weeks, preferably before or during meals, with plenty of water. Following local legislation for food supplements, the product used is the same as that available in the market and must be administered as reported in the instruction for use. No changes in the dosage are planned during the study period. However, the investigator may decide to discontinue IP administration in case of adverse events (AEs) and serious AEs (SAEs) or to use other therapies at any time if he or she deems it necessary for the patient’s health. The reason for discontinuing the administration must be documented in both the source documents and CRF. At BL, each patient is given an IP package for the entire duration of the study (60 tablets). Patients are instructed to return all IP packages to the investigator at the end of the study to ensure product accountability. The IP contains 2 ingredients: HMWHA and dry extract of Boswellia.

A medical literature analysis has shown that HMWHA has been effective in relieving knee pain in various clinical trials without side effects [12,13]. The amount of HMWHA in the IP used in this study, which is greater than 2.8 million Da, is comparable to or slightly higher than that of supplements already available on the market for viscosupplementation in OA [13] and used in clinical trials [19-21].

Among the boswellic acids obtained from the gum resin of Boswellia species, 11-keto-β-boswellic acid and 3-O-acetyl-11-keto-β-boswellic acid (AKBA) show strong evidence of downregulation of cytokine production and inhibition of inflammatory response enzymes [22]. A phase Ia study performed in the United States in 20 patients with invasive breast cancer [23] shows that oral administration of boswellia at a dose of 2400 mg daily for 5-56 days has a good safety profile, is well tolerated, and reduces breast tumor cell proliferation. A recent review [24] suggests that AKBA could be an effective and safe treatment option for patients with OA with a treatment duration of at least 4 weeks. There is a discrepancy between the amount of Boswellia extract and boswellic acid present in the tested IP (100 mg of dry extract and 10% AKBA, respectively) and the significantly higher levels found in products on the market in Europe and the United States (in particular, AKBA ranges from 17% to 66%) [25]. Furthermore, in the literature, improvement in OA symptoms has only been demonstrated in clinical trials using at least 20% AKBA [26,27].

### Concomitant Care and Interventions

For ethical reasons, we decide to administer also rescue medications. This term [28] refers to drugs that can be administered to patients in a trial if the efficacy of IP is not satisfactory.

Two boxes of Paracetamol LPH 500 (40 tablets in total) are delivered at the BL and collected at the final visit (week 8). Each tablet contains 500 mg of paracetamol. If requested by the patient, an additional box of 20 tablets is delivered at week 4. In any case, a daily dosage of >3000 mg (6 tablets per day) is not allowed.

For the duration of the pilot study, participants are prohibited from consuming alcohol, abusing drugs, following an energy-restricted diet for weight loss, taking diuretics, or any medication listed in the exclusion criteria. Additionally, routine intake of antiresorptive drugs such as bisphosphonates or estrogen is not permitted.

There are no restrictions on treatments previously administered by the participants for clinical conditions unrelated to the study.

### Objectives

The primary objective of this pilot study is to test the same outcomes that will be implemented in the future RCT. This next trial will have a calculated sample size of at least 80 patients and a study duration of 4 months. Therefore, the feasibility of using ultrasound and range of motion (ROM) to objectively measure the potential improvement in the tested joint mobility during the trial is evaluated. Additionally, we investigate whether there is a correlation between ROM or ultrasound improvement and subjective VAS scores.

This pilot study, even if it is a small-scale investigation, should also confirm the safety data belonging to the large use of oral HA in clinical practice.

Another objective is to collect data to assess the feasibility of the future main RCT in advance, estimating the time and budget required. The collected data can be both quantitative and qualitative.

Finally, this study should provide preliminary data on the efficacy of the tested product. However, these data will not be exhaustive, as the focus of the pilot study is on the reliability of the outcome and feasibility of measurement, rather than statistical significance [29].

### Potential Risks

The potential risks to patients are related to the administration of oral HA, AKBA, and the rescue medicine (paracetamol). Although oral HA has no documented side effects, some users reported skin rashes and blurred vision after oral HA administration. These reports were anecdotal. In rare cases, HA (only if administered via IA) may cause allergic reactions [11].

In rare cases, AKBA may cause life-threatening allergic reactions, which can be identified by symptoms such as chest pain, breathing difficulties, hives, or swelling. Although AKBA is generally well tolerated, it may cause stomach discomfort, nausea, heartburn, fullness, or diarrhea [30].

Considering that the rescue medication paracetamol could be used only occasionally (high doses and prolonged treatment are not allowed in this study, and pre-existing renal and hepatic impairment are in the exclusion criteria), the potential risk should be low. In any case, the possible risks of this drug include hypersensitivity reactions (transient rash, paresthesia, and pruritus), thrombocytopenia (generally asymptomatic, rarely bleeding or bruising, black and soft stools, blood in urine and feces, and red spots on the skin), agranulocytosis (pharyngitis and fever, unexpected), dermatitis, hepatic impairment (conjunctival jaundice or skin current), renal colic with sudden onset of severe lumbar pain, and renal impairment (oligoanuria).

### Outcomes

#### Primary Outcomes

1. ROM: The improvement in active and passive knee flexion and extension, measured in degrees using a goniometer and assessed by the investigator, between the BL visit and the final visit.
2. Ultrasound: A qualified physician records improvements in ultrasound parameters including synovial fluid, articular cartilage damage, medial meniscus protrusion, lateral meniscus protrusion, medial osteophytes, lateral osteophytes, enthesopathies, and effusions.
3. VAS: The improvements in VAS (at rest) scores between the BL and final visit are measured in millimeters. A paper printed scale of 100 mm was used to measure pain intensity. Patients must draw a vertical line at the point considered to be the level of pain at a specific moment, that is, resting, moving, or pressing.
4. Safety: Investigators must collect and evaluate all AEs and SAEs according to the current local legislation. AEs and SAEs must be summarized based on the number of affected patients. This includes related AEs, including those that are possibly related to or not evaluable. The incidence, type, and severity of AEs or SAEs are presented in frequency tables using MedDRA (Medical Dictionary for Regulatory Activities) terms. Frequency is compared using the χ^2^ test

#### Secondary Outcomes

Parameters necessary to estimate the time and budget required for the main study, which include the following:

1. Quantitative parameters, such as monthly recruitment rate, duration of enrollment and visits, choice of end points, and measurement.
2. Qualitative parameters, such as information on patient reluctance to enroll, difficulty understanding the consent form, logistic issues at the site, interpersonal relationships among staff, and patient-reported QoL measured by the KOOS questionnaire (paper printed version) and the Lequesne Algofunctional Index (which also includes physical activity questions).

At the end of the pilot trial, the following criteria are used to determine whether to proceed with the future main RCT: there must be evidence of improvement in both the subjective outcome (VAS at rest) and at least one of the objective outcomes (ROM and ultrasound) at the final visit. On the contrary, due to the small sample size, a statistically significant correlation (using the Pearson coefficient) between objective and subjective parameters is not required.

#### Exploratory Outcomes

In addition to the primary and secondary outcomes, we identify exploratory outcomes that could be implemented in the main trial:

1. Actigraphy: This specific tool measures physical activity levels using a triaxial accelerometer that collects 24/7 data points during the patient’s daily activities. The investigator downloads and processes the data using specific software during routine visits at the center. The instrument is available in the market and has already been tested in OA clinical trials to objectively evaluate the composite measurement of pain and basic activities such as walking or performing daily tasks [31,32]. In this pilot study, an actigraph is worn around the knee to record data for 7 days.
2. Rescue medication: Measuring the use of rescue medication during a clinical trial can provide information on the robustness of the analgesic efficacy of the tested product. Rescue medication may also be used as a primary end point according to the US Food and Drug Administration guidelines [33].

This study also provides preliminary data on the efficacy of the tested product.

### Framework of the Study

**Figure 3:**
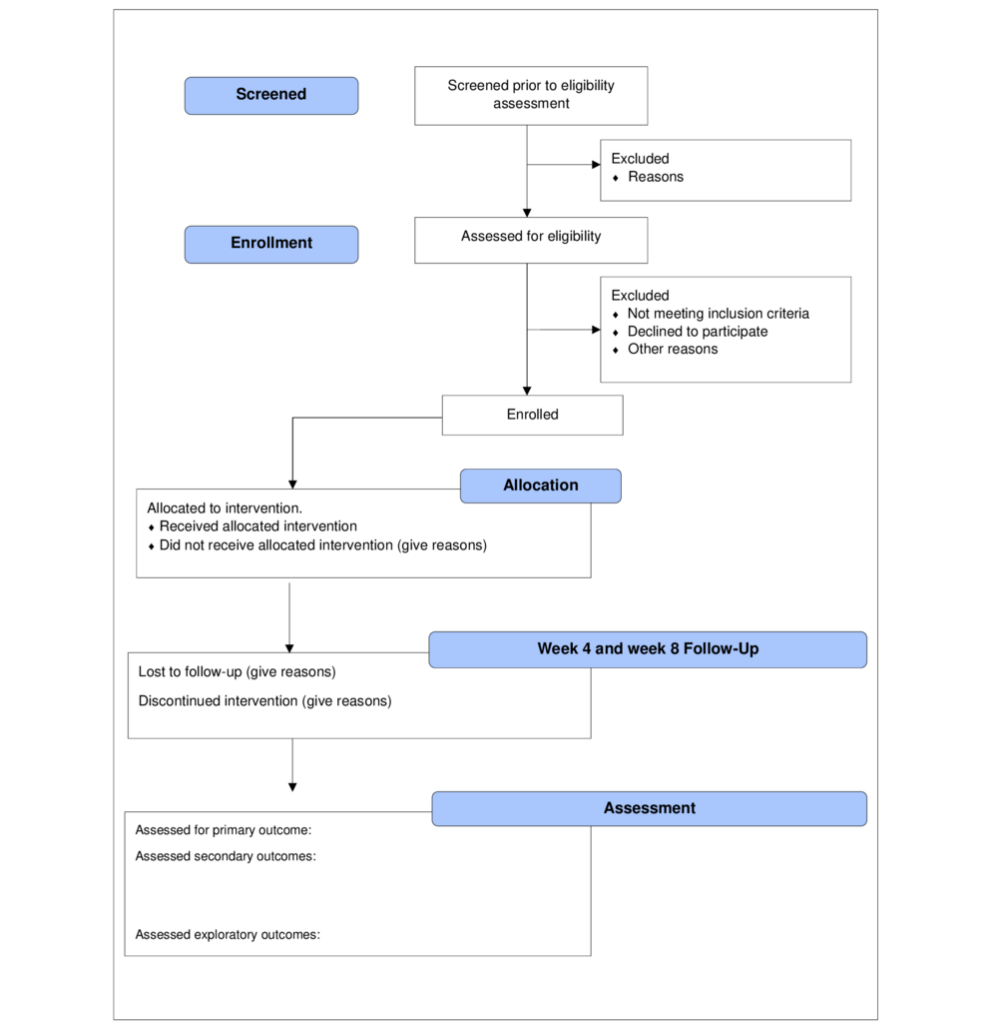
Flow diagram (CONSORT [Consolidated Standards of Reporting Trials]) of the pilot study.

Before any study procedure, patients receive information about the nature, purpose, benefits, and risks of the trial. Participants are then asked to sign an informed consent form. After the screening assessment (from day –14 to day 0), patients undergo a BL visit (day 0) to assess the inclusion and exclusion criteria and check for routine orthopedic conditions (Figure 3). To confirm the diagnosis of knee OA, the investigator evaluates an x-ray test with anteroposterior and lateral views that are no earlier than 6 months old.

At BL (Table 1), the investigator also performs an ultrasound, ROM test, VAS, and the KOOS questionnaire. The actigraph must be placed above the affected knee 7 days before the visit and removed at BL. Safety is assessed by phone at week 4 ± 2 days, or an optional visit should be conducted to complete the questionnaires, scales, and scores.

The final visit is scheduled for week 8 ± 4 days to conduct an ultrasound, ROM assessment, VAS, and the KOOS questionnaire. Patients wearing the actigraph will also be asked to return to the site 7 days before the visit to place the device above the affected knee; it will be removed during the final visit.

**Table 1:** Schedule of observation points and assessments.

Assessments	Visit 1	Visit 2	Visit 3
Days	–14 to 0	28 (±2)	56 (±4)
Weeks	–2 to 0	4	8
Informed consent	✓		
Inclusion criteria	✓		
Exclusion criteria	✓	✓	✓
Demographics and medical history	✓		
Physical examination and vital signs	✓		
Orthopedic assessment	✓		✓
Concomitant treatments	✓	✓	✓
Actigraphy—sensor delivery (1 week before the visit; optional)	✓		✓
Actigraphy—sensor return (optional)	✓		✓
KOOS^a^	✓	✓^b^	✓
VAS^c^	✓	✓^b^	✓
ROM^d^ by a goniometer	✓	✓^b^	✓
Ultrasonography	✓		✓
IP^e^ delivery	✓		
IP return			✓
Rescue medication delivery	✓		
Rescue medication return			✓
Rescue medication accountability card delivery	✓		
Rescue medication accountability card return			✓
Adverse events	✓	✓^f^	✓

^a^KOOS: Knee Injury and Osteoarthritis Outcome Score.

^b^Tests and examinations not mandatory if the visit is performed via telephone call.

^c^VAS: visual analog scale.

^d^ROM: range of motion.

^e^IP: investigational product.

^f^Safety (adverse event or serious adverse event checking) is mandatory in any case.

### Sample Size

For feasibility and pilot trials, it is important to justify the sample size; however, a formal calculation may not always be necessary [34] and is often not reported. In fact, a pilot study is not a hypothesis testing study, and often its main purpose is to test the feasibility of the proposed approach. This involves testing quantitative parameters, such as outcomes and resources, as well as qualitative factors, such as QoL and logistical issues at the site.

Although some papers suggest a sample size of 12-20 participants for pilot studies, it is generally reported that 10%-20% of the main sample size is a reasonable number for conducting such trials [35]. Given that the sample size of our main RCT will be 80 patients and the exploratory nature of this pilot study, investigators have agreed to enroll only 8 participants.

### Statistical Analysis

Quantitative variables, such as demographics, are described using mean and SD if normally distributed and median and IQR if nonnormally distributed. Comparative analysis is performed using either the Student *t* test (2-tailed) or the Mann-Whitney *U* test, depending on the distribution of the variables. Factorial variance analysis can be used to evaluate interactions between quantitative variables and linear progression models to identify possible confounding bias with independent variables. Categorical variables are described using frequencies and percentages, and comparative analyses using the χ^2^ test. The Spearman rank correlation, a nonparametric test, measures the degree of association between 2 variables. The Spearman rank correlation test is appropriate for analyzing correlations when variables are measured on at least an ordinal scale, as is the case in this study. This test does not make any assumptions about the distribution of the data. When analyzing the enrolled population, we use the Spearman rank correlation test to identify any strong positive correlations (at least 0.7) between the reduction in VAS (at rest) and ultrasound parameters, as well as between the reduction in VAS (at rest) and ROM improvement.

We evaluate the quality and completeness of the collected data before conducting any analysis. If a participant is missing information for 1 or more variables, even after the queries are resolved, the missing data are not replaced. Additionally, if a participant has violated the inclusion and exclusion criteria, the corresponding data are excluded from the analysis.

## Results

Recruitment and treatment of the 8 patients began on February 15, 2018, and was completed on May 25, 2018. Data analysis was planned to be completed by the end of 2018, with full results expected to be published in the last quarter of 2024.

## Discussion

### “You Have to Walk Before You Can Run”

We wanted to perform an RCT to test oral HMWHA in patients with OA. We calculated the sample size (approximately 80 evaluable participants), defined the design (double-blind randomized vs placebo), and estimated the duration of treatment (at least 4 months). We have previously conducted and published a systematic review of existing RCTs on HA [13]. We were even trusted to have found a potential problem that caused the limited and equivocal results of the previous RCT with HA published in the medical literature. We abruptly halted our planning and, gazing into each other’s eyes, reminded ourselves of the old adage: “You have to walk before you can run.” It would be unrealistic to attempt to build and execute a future main RCT to test oral HMWHA in OA with only the limited resources of a team of investigators in Eastern Europe. The risk of misplanning trial management (ie, duration of enrollment, sites to be involved, visits, and examinations) and the possibility of bias in the selection of outcomes is too high. It is not feasible to consider such a significant risk in terms of finances, time, and resources without first testing the project, even on a small scale. To ensure the feasibility of the monthly recruitment rate, to estimate the time and budget requirements for the main study, and to avoid potential bias in the selection of end points and their measurement, it is necessary to perform a preliminary evaluation. For this reason, the present pilot feasibility study protocol was designed and developed. Three additional issues related to trials of oral HA in patients with OA can be discussed.

The first is a comment reported by the principal investigator in a letter to the editor [36]. In particular, he reported several additional critical issues regarding the parameters used to measure the end points. The use of VAS for pain measurement and specific scores for OA outcomes, such as KOOS, as well as scales tested only in the Japanese population, for example, Japanese Knee Osteoarthritis Measure and Japanese Orthopaedic Association scales [37,38], should be considered a subjective measure despite being standardized and validated. In 7 trials conducted in Japan, only the VAS and/or other types of scales were used to measure OA symptoms, and no objective tools, such as ultrasound or isokinetic dynamometers, were used. These instruments were used in only 4 of 10 studies performed in Europe and America. However, several studies have generated ambiguous results when using ultrasound [39-41] and isokinetic dynamometers [40-42]. In only 2 studies, ultrasound measurements showed a statistically significant difference from the control group [39,40]. Furthermore, it is hard to define a positive result when the only statistically proven difference from the control group is the isokinetic peak torque at 240° of the extensors [37]. In 2 trials [39,41], the methods used to apply and evaluate ultrasound examinations were not reported. In the third study [40], the only ultrasound data collected was the measurement of synovial effusion in the suprapatellar recess on the longitudinal axis in millimeters following the 2005 EULAR guidelines [43]. Isokinetic tests were conducted using the Biodex System (Biodex Medical Systems), which is considered the gold standard for such measurements. However, in the study protocol, it was not considered that an increased level of synovial fluid (4 mm in the suprapatellar recess) could be a common symptom in patients with knee OA. Therefore, in several cases in the abovementioned trials, excess synovial fluid could cause pain and interfere with the isokinetic assessment of muscle strength [40]. The reason for using this tool as the primary efficacy assessment in 3 trials was that a reduction in knee OA pain could indicate an increase in work, power, and peak torque of the leg muscles. This surrogate end point could be useful for testing HA on joint pain in athletes or young people. In contrast, the studies that tested isokinetic assessment had patients with a mean (SD) age of 56.1 (8.00) years [40], 42.38 (10.16) years [41], and 59.6 (8.3) years [42]. Even if there were a statistically significant difference in muscular strength in a similar population of older patients, it would not be considered clinically important because this parameter does not affect the patients’ QoL.

None of the trial reviews objectively evaluated the composite measurement of pain and basic activities such as walking or performing daily tasks. Actigraphs, which are capable of recording these data, have been tested in clinical trials with patients with knee OA [32]. The cost of a single actigraph is approximately US $100, which is significantly less than that of the Biodex system. Additionally, actigraphs are user-friendly, and their data can be collected using a standard personal computer.

Second, ROM, another tool that can objectively measure the potential improvement in OA using a simple goniometer, paradoxically was not included as an outcome in these trials. ROM measurement remains widely used in orthopedics, and we believe that it can be easily correlated with specific scales such as VAS and KOOS to improve patient assessment. In this pilot study, because ROM is not the best way to assess disability in severe OA, we only included patients with a Kellgren-Lawrence score of 2.

The third topic is our willingness to include the daily average consumption of rescue medications as an exploratory outcome. In a similar OA population with a Kellgren-Lawrence score of 2 or 3 and treated with placebo for 24 weeks, the number of paracetamol 500 tablets used per day ranged from 0.34 [44] to 1.32 [33]. Effective medications should be the only option to reduce the need for rescue medications, so collecting and evaluating this objective parameter should be straightforward.

The criteria that we have identified for determining whether to proceed with the future RCT are improvement in both the subjective outcome (VAS at rest) and at least one of the objective outcomes at the final visit. We also believe that owing to the small sample size of this pilot study, a statistically significant correlation between objective and subjective parameters as a condition for starting the main future RCT is not required at this stage.

On the basis of the findings of this open-label pilot feasibility study, we will be able to design and conduct the future double-blind RCT to evaluate the efficacy of oral HA in knee OA. In clinical trials, investigators generally record these findings as the correlation between objective and subjective outcomes (ie, ROM and VAS, respectively). In daily medical practice, orthopedics uses the same findings (the link between the subjective symptoms reported by the patient and the objective signs of their visit) to demonstrate the patient’s improvement. Therefore, correlating objective and subjective improvements in patients affected by knee OA could provide an additional measurement tool for medical doctors in their daily orthopedic practice.

### Limitation of the Study

The main limitation of this study is the absence of a control group. In fact, to reduce costs and simplify the protocol, the placebo group was not included.

A second limitation is that the IP used does not contain HA alone, but a combination of HA and a very low amount of boswellic acid (10% AKBA). With such a low dose of AKBA, only 1 study [45] showed positive results for the treatment of OA. This study used a product that contains 14.4 mg of AKBA combined with a high dose of methylsulfonylmethane, a well-known SYSADOA. Therefore, it can be assumed that in the pilot study, the effect of HA should be more prevalent than that of AKBA.

Finally, the KOOS questionnaire is used in this pilot study, although the WOMAC is the most widely used test for evaluating hip and knee OA. The KOOS evaluates both the short- and long-term consequences of knee injuries. The KOOS is selected for this study as it is a free and open-access alternative to the expensive WOMAC index. The KOOS is an extension of the WOMAC.

## Appendix

Multimedia Appendix 1 Logo of the pilot study.

Multimedia Appendix 2 Ethics Committee approval.

## Abbreviations

ACR: American College of Rheumatology
AE: adverse event
AKBA: 3-O-acetyl-11-keto-β-boswellic acid
BL: baseline
CRF: case report form
CRO: Contract Research Organization
EC: ethics committee
ESCEO: European Society for Clinical and Economic Aspects of Osteoporosis, Osteoarthritis and Musculoskeletal Diseases
GCP: Good Clinical Practice
GDPR: General Data Protection Regulations
HA: hyaluronic acid
HMWHA: high-molecular-weight hyaluronic acid
IA: intra-articular
IP: investigational product
KOOS: Knee Injury and Osteoarthritis Outcome Score
MedDRA: Medical Dictionary for Regulatory Activities
OA: osteoarthritis
QoL: quality of life
ROM: range of motion
SAE: serious adverse event
SYSADOA: symptomatic slow-acting drug for OA
VAS: visual analog scale
WOMAC: Western Ontario and McMaster Universities Osteoarthritis Index

## Data Availability

The data sets analyzed during this study are available from the corresponding author upon reasonable request.
