# Supplementary figures and images for: Assessment of the Feasibility of Objective Parameters as Primary End Points for Patients Affected by Knee Osteoarthritis: Protocol for a Pilot, Open Noncontrolled Trial (:SMILE:)
Source: JMIR Res Protoc. 2024 Jun 28;13:e13642. doi: 10.2196/13642 (PMC11245663; doi:10.2196/13642)

## Slide 1
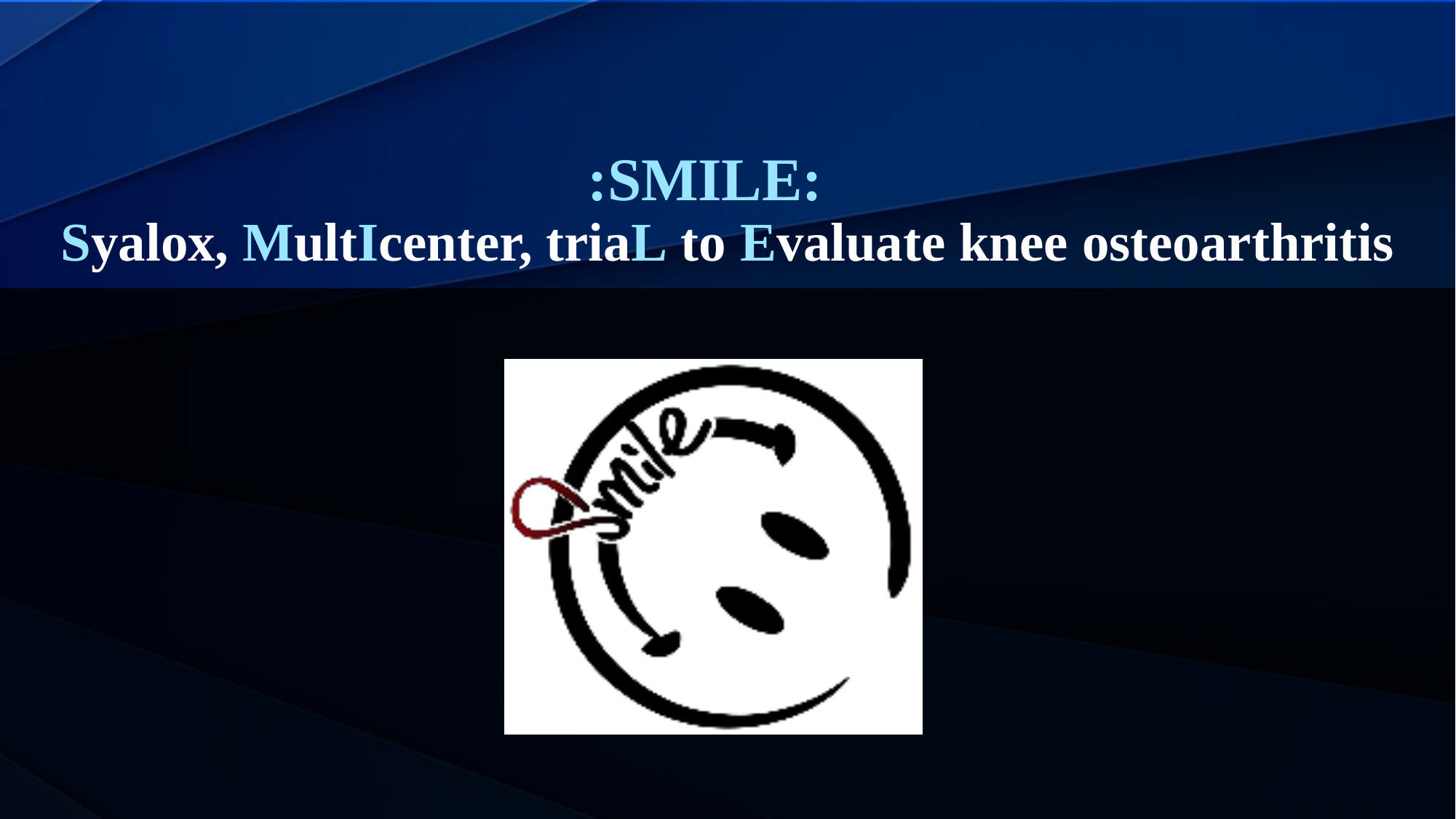

# :SMILE: Syalox, MultIcenter, triaL to Evaluate knee osteoarthritis

Supplement: Multimedia Appendix 1 [file resprot_v13i1e13642_app1.pptx]
