# Supplementary material for: Assessment of the Feasibility of Objective Parameters as Primary End Points for Patients Affected by Knee Osteoarthritis: Protocol for a Pilot, Open Noncontrolled Trial (:SMILE:)
Source: JMIR Res Protoc. 2024 Jun 28;13:e13642. doi: 10.2196/13642 (PMC11245663; doi:10.2196/13642)
Supplement: Multimedia Appendix 2 [file resprot_v13i1e13642_app2.pdf]

MEDICALI'S
Timișoara, Str. Cernauti, nr.13

Decizia Comisiei Locale de Etică pentru cercetare științifică a

Centrului MEDICALI'S

Ca răspuns la adresa Dumneavoastră, vă comunicam avizarea din punct de vedere etic al studiului clinic intitulat:

"Studiu pilot, deschis, necontrolat pentru a evalua fezabilitatea implementarii parametrilor obiectivi ca scop primar într-un studiu clinic cu pacienții afectați de osteoartrita la nivelul genunchiului"
Număr de protocol: OPRPH/0117/FS, Versiune: 1.0 finală din 05.12.2017

Investigator Principal: ***Dr. Bogdan Corneliu Andor***

Comisia Locală de Etică pentru cercetare științifică a Centrului MEDICALI'S funcționează în conformitate cu prevederile art. 167 din Legea nr. 95/2006, art. 28, cap. VIII din ordinul 904/2006, Directivei 2001/20/EC A Parlamentului European și a consiliului din 4 aprilie 2001 și cu Declarația de la Helsinki –editia 64th emisă de OMS la Fortalezza, Brazilia, Octombrie 2013.

În urma analizei documentelor transmise, Comisia de Etică avizează favorabil desfașurarea studiului clinic sus-mentionat.

Cu stimă,

Dr. Costea Daniel - Președinte Comisia de Etică

Dr. Fratila Mihaela - Membru Comisia de Etică

Av. Bizera Ruxandra - Membru Comisia de Etică

Terteci Janina - Membru Comisia de Etică

Militaru Cristina - Membru Comisia de Etică

Timișoara, Data 12.12.2017
